# The Default Mode Network and EEG Regional Spectral Power: A Simultaneous fMRI-EEG Study

**DOI:** 10.1371/journal.pone.0088214

**Published:** 2014-02-05

**Authors:** Irene Neuner, Jorge Arrubla, Cornelius J. Werner, Konrad Hitz, Frank Boers, Wolfram Kawohl, N. Jon Shah

**Affiliations:** 1 Institute of Neuroscience and Medicine 4, INM 4, Forschungszentrum Jülich, Jülich, Germany; 2 Department of Psychiatry, Psychotherapy and Psychosomatics, RWTH Aachen University, Aachen, Germany; 3 JARA – BRAIN – Translational Medicine, RWTH Aachen University, Aachen, Germany; 4 Department of Neurology, RWTH Aachen University, Aachen, Germany; 5 Department of Psychiatry, Psychotherapy and Psychosomatics, Psychiatric University Hospital Zurich, Zurich, Switzerland; National Research & Technology Council, Argentina

## Abstract

Electroencephalography (EEG) frequencies have been linked to specific functions as an “electrophysiological signature” of a function. A combination of oscillatory rhythms has also been described for specific functions, with or without predominance of one specific frequency-band. In a simultaneous fMRI-EEG study at 3 T we studied the relationship between the default mode network (DMN) and the power of EEG frequency bands. As a methodological approach, we applied Multivariate Exploratory Linear Optimized Decomposition into Independent Components (MELODIC) and dual regression analysis for fMRI resting state data. EEG power for the alpha, beta, delta and theta-bands were extracted from the structures forming the DMN in a region-of-interest approach by applying Low Resolution Electromagnetic Tomography (LORETA). A strong link between the spontaneous BOLD response of the left parahippocampal gyrus and the delta-band extracted from the anterior cingulate cortex was found. A positive correlation between the beta-1 frequency power extracted from the posterior cingulate cortex (PCC) and the spontaneous BOLD response of the right supplementary motor cortex was also established. The beta-2 frequency power extracted from the PCC and the precuneus showed a positive correlation with the BOLD response of the right frontal cortex. Our results support the notion of beta-band activity governing the “status quo” in cognitive and motor setup. The highly significant correlation found between the delta power within the DMN and the parahippocampal gyrus is in line with the association of delta frequencies with memory processes. We assumed “ongoing activity” during “resting state” in bringing events from the past to the mind, in which the parahippocampal gyrus is a relevant structure. Our data demonstrate that spontaneous BOLD fluctuations within the DMN are associated with different EEG-bands and strengthen the conclusion that this network is characterized by a specific electrophysiological signature created by combination of different brain rhythms subserving different putative functions.

## Introduction

Resting state network (RSN) activity can be defined as coherent and spontaneous fluctuations of human brain activity in distinct and spatially separate networks of varying granularity when subjects are not engaged in a particular task or superior cognitive processes. The concept was developed as a consequence of evaluating the functional connectivity among brain regions displaying spontaneous functional magnetic resonance (fMRI) activity recorded at rest [1]–[4]. Biswal *et al.* took the first steps when they demonstrated a high correlation and temporal synchrony of the blood oxygenation level dependent (BOLD) contrast in series of relatively distant brain regions [5]. The existence and characteristics of the RSNs have been further studied in a series of magnetic resonance imaging and positron emission tomography studies [6]–[8] and their abnormalities have been related to neurological and psychiatric conditions [9]. Changes in the connectivity and patterns of the RSNs have also been described during normal human sleep [10], [11]. Additionally, RSN changes have been described in normal aging [12]. The importance of RSN activity is also highlighted by the physiological energy demand of the brain during “rest”: 60% to 80% of the brain's energy usage is used to support communication among neurons and basal activity; the additional energy burden associated with transient demands such as evoked activity is as little as 0.5% to 1% of the total energy [13].

One of the most frequently studied and robustly measurable RSN is the default mode network (DMN), which is thought to characterize basal neural activity [13]–[15]. It comprises the precuneus, anterior cingulate cortex (ACC), posterior cingulate cortex (PCC) and lateral parietal inferior gyri [9], [16], [17]. The activity of the DMN has been linked to introspection, self-referential thought and integration of cognitive and emotional processing [2]. RSNs are referred to as “low frequency” signals in reference to their spectral power distribution [18]. The actual neuronal basis of the low frequency BOLD signal oscillations is not completely understood and has spawned a debate about the possibility that these BOLD signals arise from respiratory or cardiac oscillations [19]–[23].

In contrast to the indirect character of the BOLD signal, electroencephalography (EEG) is a direct measure of neuronal activity, and provides an effective means of measuring neuronal firing [24], [25]. It requires the synchronous activity of a large number of neurons to generate measurable electric potentials at the scalp. It thereby poses the problem of source localization (an inverse problem), in which only surface measurements are made of signals originating inside a conductive volume [26]. In this sense, simultaneous fMRI-EEG has gained attention due to the complementary temporal and spatial resolutions inherent to each technique [27]. This simultaneous multimodal approach has the potential to control for confounding factors such as the arousal level and the differences of the physiological state at rest.

The relationship between EEG and fMRI signals is still a topic of ongoing research. Based on a study in anaesthetised monkeys undergoing fMRI and microelectrode recordings by Logothetis *et al.*, it was suggested that the BOLD signal is governed by local field potentials [28]. However, the exact mechanism of coupling between the hemodynamic response measured by BOLD-fMRI and the underlying neuronal activity is poorly understood and is an area of intensive discussion and research [6], [29], [30].

Previous simultaneous fMRI-EEG studies have focused on the alpha and beta frequency bands and they suggest that the BOLD signal in visual regions and other cortical areas is negatively correlated with posterior alpha fluctuations observed in the EEG data [25], [31]. Laufs *et al.* observed that “positive correlation with alpha power (and the BOLD signal) was sparse and restricted to two foci in the cingulate gyrus and occipital cortex”, although they observed a “widespread negative correlation with alpha power in a bilateral fronto-parietal network” [31]. For the beta-band, Laufs *et al.* report positive correlations with structures belonging to the DMN network [25], [31]. Moosmann *et al.* reported, in a combined fMRI-EEG and near infrared spectroscopy (NIRS) study, an inverse relationship between alpha activity and BOLD signal in the occipital cortex. The NIRS-EEG results showed a positive correlation in the occipital cortex between alpha activity and fluctuations of deoxygenated haemoglobin with a temporal shift of about 8 seconds. Moosmann *et al.* proposed that alpha activity in the occipital cortex is associated with metabolic deactivation [32]. Furthermore, Goldman *et al.* showed in an early fMRI-EEG study at 3 T, that increased alpha-band power was correlated with decreased BOLD signal in the occipital, superior temporal, inferior frontal and cingulate cortex, and with increased signal in the thalamus and insula [33].

Several other studies have investigated the functional link between EEG oscillations and RSNs using different approaches. Mantini *et al.* concluded that the sensorimotor RSN is primarily associated with beta-band oscillations, and that the visual RSN is associated with all frequency bands except gamma [34]. Jann *et al.* proposed that EEG frequency bands and their topographies can be seen as electrophysiological signatures of the underlying distributed neuronal RSNs, and they reported that the activity of DMN was associated with increased alpha activity in occipital electrodes and beta activity in parietal electrodes [35]. Knyazev *et al.* found, via independent component analysis (ICA), that only the spatial patterns of the alpha-band showed an overlap with the DMN, suggesting that the primary function of alpha oscillations is the synchronization of internal mental processes [36]. In a recent study, Sadaghiani *et al.* [37] used simultaneous fMRI-EEG to analyse the relationship between a given frequency band in EEG and the BOLD signal, although focusing on phase synchronization. Based on the hypothesis that alpha-band oscillations function as an “active inhibitory mechanism that gates and controls sensory information processing”, a positive correlation was found between upper alpha-band phase synchrony and the BOLD signal in prefrontal and parietal regions [37].

ICA in fMRI data analysis has been demonstrated to be a reliable tool for identifying patterns of activation, image artefacts and RSNs [14], [38], [39]. The resulting activation maps that correspond to RSNs differ significantly from the maps of major blood vessels and motion artefacts. Additionally, the method of ‘dual regression’ was recently introduced, which permits the identification of inter-subject differences in resting functional connectivity based on inter-subject similarities within the framework of multi-subject-ICA analysis [40].

The present study investigates EEG power correlates of the BOLD signal arising in the DMN in simultaneous fMRI-EEG using ‘dual regression’ as method for assessing inter-subject variations. In order to investigate whether different EEG frequency bands correlate with the DMN, frequency and source localization analyses were performed. We hypothesize that different frequency bands correlate with BOLD signal fluctuations of the brain at rest within the DMN.

## Materials and Methods

### Subjects

During a single session measurement, EEG was recorded simultaneously with fMRI at 3 T from 15 healthy volunteers (10 males, 5 females, mean age: 28.26 years SD: 7.35) using MR compatible devices. Written, informed consent was obtained from all subjects and the study was approved by the Ethics Committee of the Medicine Faculty of the Rheinisch-Westfälischen Technischen Hochschule Aachen (RWTH Aachen University). The study was conducted according to the Declaration of Helsinki.

EEG data were recorded by Brain Vision Recorder (Brain Products, Gilching, Germany) using a 64-channel MR compatible EEG system including an MR compatible amplifier and a synchronisation box (Brain Products, Gilching, Germany). The EEG cap (BrainCap MR, EasyCap GmbH, Breitbrunn, Germany) consisted of 63 scalp electrodes distributed according to the 10–10 system and one additional electrode for recording the electrocardiogram (ECG). Data were recorded relative to an Fpz reference and a ground electrode that was located at AFz (10–5 electrode system) [41]. Data were sampled at 5000 Hz, with a bandpass of 0.016–250 Hz. Impedances at all recording electrodes were kept below 10 kΩ.

Functional MRI data were recorded using a 3 T Siemens Magnetom Trio scanner. Images were acquired using a T2*-weighted EPI sequence (TR: 2.2 s, TE: 30 ms, FOV: 200 mm, slice thickness: 3 mm and number of slices: 36). The functional time series consisted of 165 volumes (6 minutes). Anatomical images were acquired for every subject using a Magnetization-Prepared, Rapid Acquisition Gradient-Echo (MP-RAGE) sequence (TR: 2250 ms, TE: 3.03 ms, field-of-view: 256×256×176 mm^3^, matrix size: 256×256, flip angle: 9°, 176 sagittal slices with 1 mm slice thickness and GRAPPA factor of 2 with 70 autocalibration signal lines). The subjects were requested to lie down, close their eyes and relax during the six minutes of measurement.

### EEG data analysis

The EEG data were processed using Brain Vision Analyzer (Version 2.0. Brain Products, Munich, Germany). Gradient artefact correction was performed using the method proposed by Allen *et al.* and included in Brain Vision Analyzer [42]. Data were down-sampled to 250 Hz and a low-pass filter with a cut-off frequency of 40 Hz was applied. Re-referencing of the data was carried out including all EEG channels as a new reference. Pulse artefact correction was achieved using ICA [43], [44]. In order to obtain independent components (ICs), the extended Infomax ICA [45] with the Runica algorithm was applied to the whole data set. The pulse artefact correction was accomplished by visual identification of components contributing to the artefact and rejecting them using the ‘Inverse ICA’ tool. ‘Complex demodulation’ was performed in order to extract frequency power at each data point. The complex demodulation works by transforming the EEG signal continuously in such a way that the resulting signal consists only of frequencies that lie within in the defined range [46]. Complex demodulation is a local version of harmonic analysis that enables the amplitude and phase of particular frequency components of a time series to be described as functions of time.

The frequency bands were defined as follows: delta (0.5–3.5 Hz), theta (4–7 Hz), alpha-1 (7.5–9.5 Hz), alpha-2 (10–12 Hz), beta-1 (13–23 Hz) and beta-2 (24–34 Hz), according to the conventional International Federation of Clinical Neurophysiology guideline [47]. For the construction of covariants, EEG power was extracted from regions-of-interest (ROI) using the Low-Resolution Electromagnetic Tomography (LORETA) [48] tool included in Brain Vision Analyzer. LORETA uses a three-shell spherical head model registered to a standardized stereotactic space available as digitized MRI data from the Brain Imaging Centre (Montreal Neurological Institute, MNI305) [49]–[51]. Registration between spherical and realistic head geometry used EEG electrode coordinates as implemented in LORETA [52]. The cortical structures comprising the DMN were chosen as anatomic ROIs: ACC, PCC, precuneus and lateral parietal inferior gyri. All ROIs were extracted according to the Talairach atlas coordinates of the Montreal Neurological Institute's MRI average of 305 brains [51]. Time courses of EEG activity within these ROIs were exported as mean activity and used as covariants in the analysis of the fMRI data. In order to give more validity to the approach, an additional source localisation analysis of the alpha-band oscillations (7.5–12 Hz) was performed using LORETA in search of an occipital source for these oscillations [53].

Additionally, sleep scoring was performed for each individual using pulse artefact corrected data. This was accomplished according to the visual scoring of sleep in adults of the American Academy of Sleep Medicine [54]. The data from all 15 subjects were segmented in 30 seconds epochs and visually inspected in search of alpha attenuation in the occipital electrodes. Furthermore slow eye movements and K-complexes were sought in frontal channels in order to define the wake-sleep stages [54].

### Functional MRI data analysis

**Figure 1:**
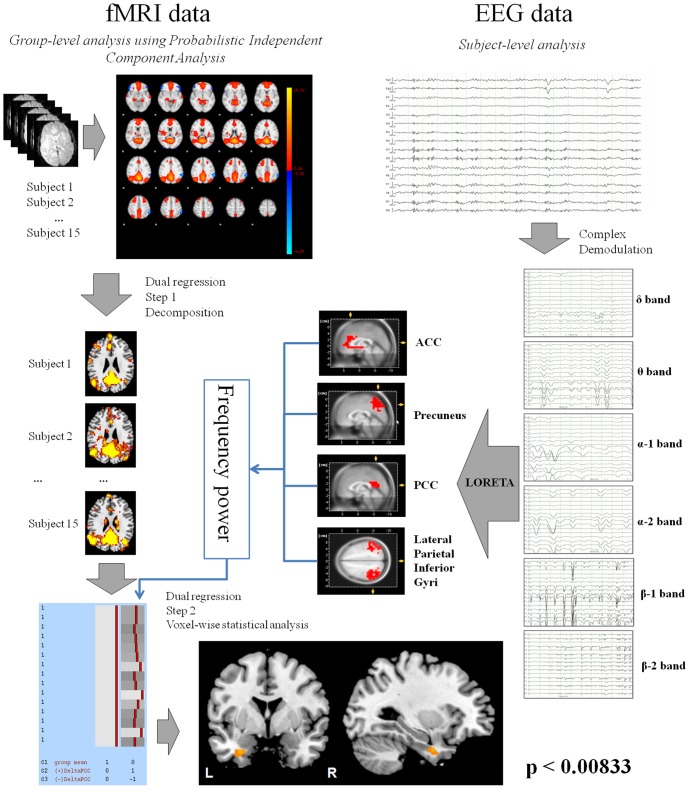
Methodological approach: LORETA analysis was performed after correction and demodulation for a specific frequency. The extracted average power was included into the dual regression analysis in order to identify brain structures whose BOLD signal variation correlates with the power of EEG-frequencies. The image below shows voxels in which alpha power extracted from the ACC was positively correlated (p<0.00833) with the activity of the DMN.

Functional data were analysed using Multivariate Exploratory Linear Optimized Decomposition into Independent Components (MELODIC), included in FSL (FMRIB's Software Library, www.fmrib.ox.ac.uk/fsl/). Data pre-processing per subject consisted of motion correction, brain extraction, spatial smoothing using a Gaussian kernel with a full-width at half maximum (FWHM) of 6 mm, and high-pass temporal filtering of 100 s. FMRI volumes were registered to the individual's structural scan and standard space (MNI152) images using FMRIB's Nonlinear Image Registration. Temporal concatenation ICA was performed across all functional datasets from each subject using automatic dimensionality estimation. The resulting ICA maps were thresholded at a mixture-modelling p<0.5. The DMN was identified by visual inspection and comparison to previously published data [16], [55]. Finally, the dual regression algorithm [40] was applied to the ICs in order to identify the individual contribution of every subject to the resting state networks using the spectrum EEG regional power as a covariant in the dual regression analysis. Here, the individual's frequency powers, sampled in the regions stated above, were tested for correlation with the individual's z-values of the IC representing the DMN. The different component maps were collected across subjects into single 4D files and tested voxel-wise for statistically significant correlation using nonparametric permutation testing (10000 permutations) [56]. This resulted in spatial maps characterizing the voxels whose connection strength within or to the DMN correlates with the EEG regional power at each frequency. In order to correct for multiple comparisons across voxels and maps, the IC maps were thresholded using the local false discovery rate (FDR) method tolerant to dependency [57] at p<0.05. The FDR threshold itself was again corrected for multiple comparisons using the Bonferroni correction method, resulting in a final significant threshold of p set to <0.00833 (0.05 divided by 6 due to six frequency bands tested). A graphic representation of this method is presented in Figure 1.

## Results

Sleep scoring of the EEG data recorded from the volunteers revealed that none of them fulfilled criteria for sleep boundary [54].

**Figure 2:**
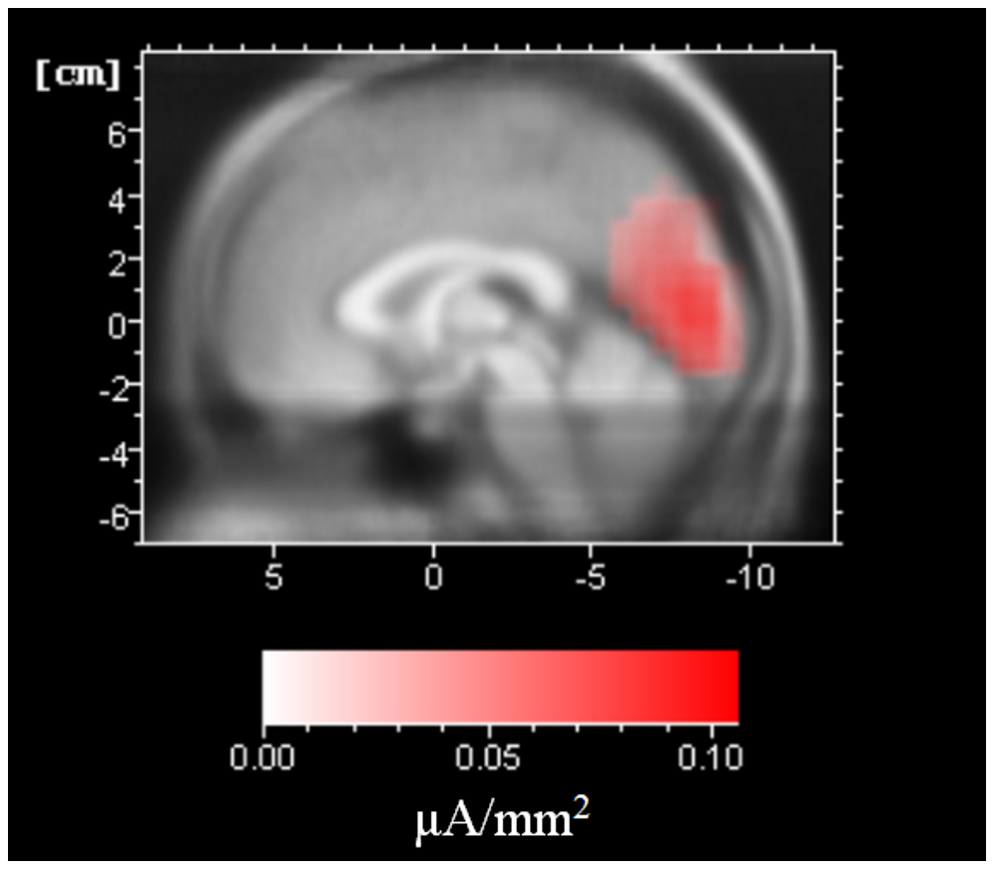
Alpha-band source localisation from the averaged signal across the 15 subjects using LORETA.

Source localisation of the alpha-band using LORETA confirmed an occipital source in all subjects. An average of the signal across the volunteers is presented in Figure 2.

**Figure 3:**
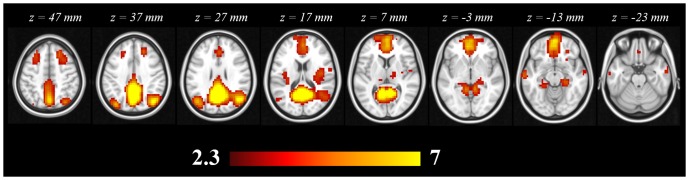
DMN identified from the group analysis of the 15 subjects.

Twenty-six independent components were found after decomposition of the functional data by means of ICA; 10 were neurologically meaningful and 16 were related to noise, head motion and vascular artefacts. The DMN was identified by visual inspection as the network comprising the ACC, precuneus, PCC and lateral parietal inferior gyri [16], [55] (Figure 3).

The statistical correlation maps display voxels with significant correlation values (p<0.00833) with the power of delta, beta-1 and beta-2 frequencies and spontaneous BOLD signal in the DMN in several areas in the brain. There were no significant correlations between BOLD signal in the DMN and power of the theta, alpha-1 and alpha-2 frequencies after FDR correction.

**Figure 4:**
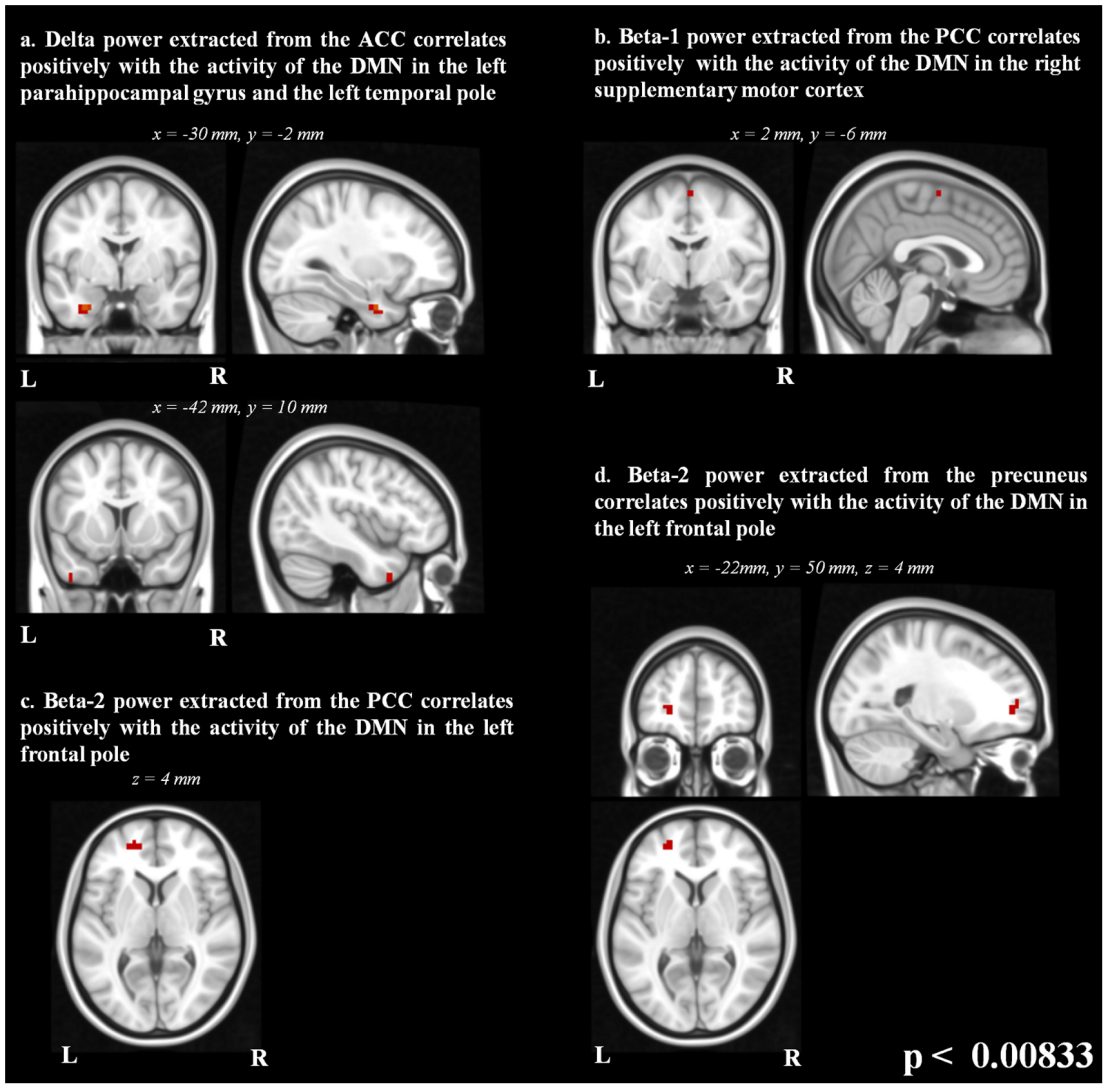
Statistical correlation maps of the EEG frequencies with the DMN signal. Significant voxels thresholded at p<0.00833 for (a) delta-band extracted from the ACC (b) beta-1 frequency extracted from the PCC, (c) beta-2 frequency extracted from the PCC and (d) beta-2 frequency extracted from the precuneus.

The delta power extracted from the ACC showed voxels with positive correlation with the DMN signal in the left parahippocampal gyrus and the left temporal pole (Figure 4, a).

The beta-1 frequency power extracted from the PCC showed voxels with positive correlation with the DMN signal in the right supplementary motor cortex (Figure 4, b).

The beta-2 frequency power extracted from the PCC and the precuneus showed voxels with positive correlation with the DMN signal in the left frontal pole (Figure 4, c–d).

All results are summarized in Table 1.

**Table 1:** MNI coordinates of clusters with minimal correlation p values with the DMN signal. Structures defined according to the Harvard-Oxford Cortical Structural Atlas.

EEG frequency and area	Regions of minimum p value according to the Harvard-Oxford Cortical Structural Atlas	Min. p value	MNI coordinates		
			*x*	*y*	*z*
Delta extracted from the ACC	34% Left Parahippocampal Gyrus, anterior division, 6% Left Temporal Fusiform Cortex, anterior division, 2% Left Temporal Fusiform Cortex, posterior division	0.005	−30	−2	−32
	77% Left Temporal Pole	0.008	−42	10	−40
Beta-1 extracted from the PCC	60% Right Juxtapositional Lobule Cortex (formerly Supplementary Motor Cortex), 7% Left Juxtapositional Lobule Cortex (formerly Supplementary Motor Cortex), 3% Right Precentral Gyrus	0.004	2	−6	64
Beta-2 extracted from the PCC	16% Left Frontal Pole	0.001	−22	50	4
Beta 2 extracted from the precuneus	16% Left Frontal Pole	0.003	−22	50	4

## Discussion

A simultaneous fMRI-EEG study was performed at 3 T in healthy volunteers to investigate the relationship between the BOLD signal of the DMN and the power of EEG frequency bands originating from within anatomical components of the DMN. To this end, we used a combination of dual regression analysis (MELODIC) for fMRI and Low Resolution Electromagnetic Tomography (LORETA) for EEG data. A strong correlation between the activity of the left parahippocampal gyrus to the DMN and the delta-band extracted from the ACC was found. Furthermore a positive correlation between the beta-1 frequency power extracted from the PCC and the connectivity values of the right supplementary motor cortex was established. In addition, the beta-2 frequency power extracted from the PCC and the precuneus showed a positive correlation with DMN connectivity to right frontal cortex. Furthermore, we verified that such correlations were not due to sleep, since the arousal state of our volunteers was checked via EEG using the visual scoring of sleep in adults of the American Academy of Sleep Medicine [54].

We consider the source localisation approach as adequate due to the fact that there was a clear occipital source of the alpha-band during eyes closed resting state [53], also suggesting that the artefact correction of the EEG data permitted a successful application of LORETA (Figure 2).

EEG frequencies have been linked to specific functions as an “electrophysiological signature” of a function, and on the other hand, a combination of oscillatory rhythms that has been related to specific functions, with or without predominance of one specific frequency band. In this sense, gamma-band oscillations have been related to attention, stimulus selection and integration, movement preparation, memory formation and conscious awareness [58]–[63]. Delta-band has been linked to learning, motivation and reward processes [64], [65], as well as to memory encoding and retrieval [66]. The activity of theta-band has been linked to working memory, emotional arousal and fear conditioning [64]. The frontal middle theta has been observed during various cognitive tasks requiring attention or working memory [67]. The alpha-band has been associated with working memory functions and short-term memory. The role of activity in the beta-band, however, has been less clear and is less well understood. In a comprehensive review, Engel and Fries suggested that beta-band activity might be associated to maintenance of motor sets and cognition [68].

The highly significant correlation between delta power within the DMN and the parahippocampal gyrus is in line with the association of delta frequencies with memory processes. We assumed “ongoing activity” of the volunteer during “resting state” in bringing events from the past to the mind, wherein the parahippocampal gyrus is a relevant structure [69], [70]. Hlinka *et al.* reported that in an inter-subject experimental design, a strong relationship was established between functional connectivity in the DMN and delta power.

In addition, a robust positive correlation between functional connectivity in the DMN and beta power was demonstrated [71]. Maintenance of the “status quo” modulated by beta-band activity has been proposed for the sensorimotor system and for cognition. It has also been proposed as a function of the DMN. Engel *et al.* hypothesized that resting state networks should be distinguished by beta frequencies [59]. Our multimodal data corroborate these proposed functions. They show a significant correlation between beta-band power extracted from the PCC with the frontal pole providing a mutual link on how the DMN sets the status quo via frontal input for cognitive functions.

According to the review by Engel *et al.* “enhancement or decrease of beta-band activity may relate not only to the involvement of top-down processing but also to the contents of the top-down signal: beta-band activity may be enhanced if the status quo is given priority over new signals” [59]. The link to the motor system is displayed in our data by significant correlations between beta-band power extracted from the PCC and the supplementary motor area (SMA). Engel *et al.* describe the role of beta-band activity not in the sense of signalling in the absence of movement but in indicating the sensorimotor system in order to maintain the current motor settings.

Our results also suggest that the DMN associates with different EEG bands as reported by Jann *et al.* [35] and supports the conclusion of Mantini *et al.* according to which a functional network is characterized by a specific electrophysiological signature created by the combination of different brain rhythms [34].

## Conclusions

In summary, we show that separate frequency domains originating in distinct subsets of the DMN are correlated with equally distinct connectivity patterns of the DMN to other cortical areas. This again highlights the multi-modal role of the DMN, which therefore cannot be regarded as a homogeneous entity. We propose fMRI-EEG studies using MELODIC/dual regression and LORETA as suitable tools to examine these connections in the normal brain and in other conditions such as ageing and neuropsychiatric diseases.
